# Green Tea Polyphenols Coupled with a Bioactive Titanium Alloy Surface: In Vitro Characterization of Osteoinductive Behavior through a KUSA A1 Cell Study

**DOI:** 10.3390/ijms19082255

**Published:** 2018-08-01

**Authors:** Martina Cazzola, Sara Ferraris, Francesco Boschetto, Alfredo Rondinella, Elia Marin, Wenliang Zhu, Giuseppe Pezzotti, Enrica Vernè, Silvia Spriano

**Affiliations:** 1 Department of Applied Science and Technology, Politecnico di Torino, C.so Duca degli Abruzzi 24, 10129 Torino, Italy; sara.ferraris@polito.it (S.F.); enrica.verne@polito.it (E.V.); silvia.spriano@polito.it (S.S.); 2 Ceramic Physics Laboratory, Kyoto Institute of Technology, Sakyo-ku, Matsugasaki, Kyoto 606-8126, Japan; boschetto.cesc@gmail.com (F.B.); al.rondinella@gmail.com (A.R.); marin_elia@hotmail.com (E.M.); wenlzhu@hotmail.com (W.Z.); pezzotti@kit.ac.jp (G.P.); 3 Department of immunology, Kyoto Prefectural University of Medicine, Kamigyo-ku, Kyoto 602-8566, Japan; 4 Department of Dental Medicine, Graduate School of Medical Science, Kyoto Prefectural University of Medicine, Kamigyo-ku, Kyoto 602-8566, Japan; 5 Department of Molecular Cell Physiology, Graduate School of Medical Science, Kyoto Prefectural University of Medicine, Kamigyo-ku, Kyoto 602-8566, Japan; 6 The Center for Advanced Medical Engineering and Informatics, Osaka University, Yamadaoka, Suita, Osaka 565-0871, Japan; 7 Department of Orthopedic Surgery, Tokyo Medical University, 6-7-1 Nishishinjuku, Shinjuku-ku, Tokyo 160-0023, Japan

**Keywords:** titanium alloy, polyphenols, green tea, bioactivity, osteoinduction

## Abstract

A chemically-treated titanium alloy (Ti6Al4V) surface, able to induce hydroxyapatite precipitation from body fluids (inorganic mineralization activity), was functionalized with a polyphenolic extract from green tea (tea polyphenols, TPH). Considering that green tea polyphenols have stimulating effects on bone forming cells (biological mineralization), the aim was to test their osteoinductive behavior due to co-operation of inorganic and biological mineralization on mesenchymal stem cells KUSA A1. The functionalized surfaces were characterized by using the Folin–Ciocalteu method and X-ray photoelectron spectroscopy to confirm the successful outcome of the functionalization process. Two cell cultures of mesenchymal stem cells, KUSA A1 were performed, with or without osteoinductive factors. The cells and surfaces were characterized for monitoring cell viability and hydroxyapatite production: Fourier Transform Infrared Spectroscopy and Raman spectroscopy analyses showed deposition of hydroxyapatite and collagen due to the cell activity, highlighting differentiation of KUSA A1 into osteoblasts. A higher production of extracellular matrix was highlighted on the functionalized samples by laser microscope and the fluorescence images showed higher viability of cells and greater presence of osteocalcin in these samples. These results highlight the ability of polyphenols to improve cell differentiation and to stimulate biological mineralization, showing that surface functionalization of metal implants could be a promising way to improve osteointegrability.

## 1. Introduction

Natural polyphenols extracted from plants have attracted increasing interest in recent years, not only in folk medicine, but also within the scientific community. Polyphenols are molecules derived from the secondary metabolism of plants and their effects on human health are widely reported in literature. They can be obtained from abundant natural sources and even from byproducts of the food/beverage industry with low costs, thereby using local resources in a sustainable way. The leaves of the tea plant (*Camellia Sinensis*), consumed in China for over 5000 years [1], are rich in extractable polyphenols which make up around 10–15% of the weight of the plant and more in specific flavan-3-ols, commonly named catechins [2,3]. They have anti-inflammatory, antioxidant, anticancer, and antibacterial effects [1,2,4,5], as well as effects on bone health by improving osteoblast mineralization and differentiation [6,7]. However, the effects of polyphenols are dose dependent and their bioavailability is a crucial issue. A large quantity of polyphenols ingested by diet cannot overcome the intestinal wall [8] and some studies demonstrate that digestion and metabolism of these molecules alter their biological properties [9,10,11], which is why local dosing of these drugs is of great interest. Surface functionalization of materials for bone contact applications could represent a promising way to stabilize polyphenols and administer them in loco in order to improve osteointegrability.

Despite extensive research on the biological properties of pure polyphenols molecules and their natural mixtures, few attempts to couple these kinds of substances with inorganic materials are reported in the literature. Natural polyphenols and pyrogallol have been coupled with steel surfaces [12]; rutin was absorbed on MCM-41 mesoporous silica nanoparticles [13] and resveratrol, quercetin, benzoic, caffeic, coumaric, ferulic and syringic acids were used for the functionalization of nanoparticles with the use of synthetic spacers [14,15,16]. Preliminary experimentation has been performed to couple polyphenols with titanium oxide layer using gallic acid esters [17], natural polyphenols and pyrogallol [12], molecules isolated from potatoes and apples [18], quercetin [19], lignin [20] and flavonoids [21]. It is of great interest to use biomaterials with inorganic bioactive behavior (hydroxyapatite spontaneous precipitation from ions in body fluids) as substrates for functionalization, in order to combine biological and inorganic mineralization abilities on the same surface. Silica-based bioactive glass and ferromagnetic bioactive glass were functionalized with polyphenols extracted from red grape skins and green tea leaves in previous works by the authors [22,23,24,25,26]. The effects of functionalization with polyphenols on bioactive glasses against cancer cells and with respect to antioxidant activity were already proved in a previous work by the authors [22]. The present paper is focused on the functionalization of a metal substrate and on its biological mineralization ability.

Ti6Al4V, a titanium alloy is widely used in dentistry (implants, miniplates and mini-screws) [27,28,29] and for the component of joint prosthetic implants that is in contact with bone [30,31]. For these applications, osteointegration is crucial and for this reason this material was selected as substrate for the functionalization.

The aim of the present research is to use natural extracts of green tea leaves for surface functionalization of a titanium alloy (Ti6Al4V), which has been chemically pre-treated. The chemical pre-treatment enriches the surface of the titanium alloy in hydroxyl groups, which allow surface functionalization and induces a bioactive behavior (spontaneous precipitation of hydroxyapatite from inorganic body fluids) [32,33]. The aim of the research is to obtain enhanced osteointegration by enriching this surface with polyphenols in order to get a synergistic action of inorganic and biological mineralization for bone healing. This treatment is designed for dental implants and osteo-integrable joint prosthetic components. In this work, the effects of functionalization with polyphenols were tested on mesenchymal stem cells, KUSA A1. After functionalization, the presence and activity of the polyphenols on the surface of the titanium alloy were checked by means of X-ray photoelectron spectroscopy analysis and spectroscopic analysis with the Folin–Ciocalteu test. After establishing a successful outcome for the functionalization procedure, the cell cultures were performed on the surface of the samples with or without the addition of further osteo-inductive agents and the cells were characterized by means of Fourier Transform Infrared Spectroscopy, RAMAN spectroscopy and fluorescence microscopy analysis. The formation of an extracellular matrix by cells was quantitatively investigated by laser microscope.

## 2. Results

### 2.1. Detection of Polyphenols

Photometric measurements were performed on the functionalized samples with the Folin–Ciocalteu method and an amount of 1.1 × 10^−3^ ± 1 × 10^−4^ mg/mL gallic acid equivalent was found on the samples after the functionalization procedure. Catechins are the main category of polyphenols in green tea. A detailed analysis of the quantification of the different polyphenols present in the extract is still ongoing.

The effect of functionalization was also checked by X-ray photoelectron spectroscopy (XPS) analysis with the acquisition of both the survey spectra, for quantitative chemical analysis, and high-resolution spectra of the carbon and oxygen regions, in order to investigate the exposed functional chemical groups. The atomic percentages of the elements revealed on the chemically-treated titanium alloy samples (named CT) and on the CT samples functionalized with TPH (named CT + TPH) samples are reported in Table 1.

First, in Table 1 it can be observed that the amount of Ti on the surface drops close to zero after functionalization. A certain amount of carbon was also detected on the CT samples (before functionalization); this could be associated with the unavoidable hydrocarbon contamination [34,35]. On the other side, the percentage of carbon on the surface of the sample CT + TPH increased significantly (4 times). Oxygen was present on the surface of the CT samples, while it was lower on the functionalized samples. Calcium was also present on the surface of the CT + TPH samples, coming from the simulated body fluid (SBF).

**Figure 1:**
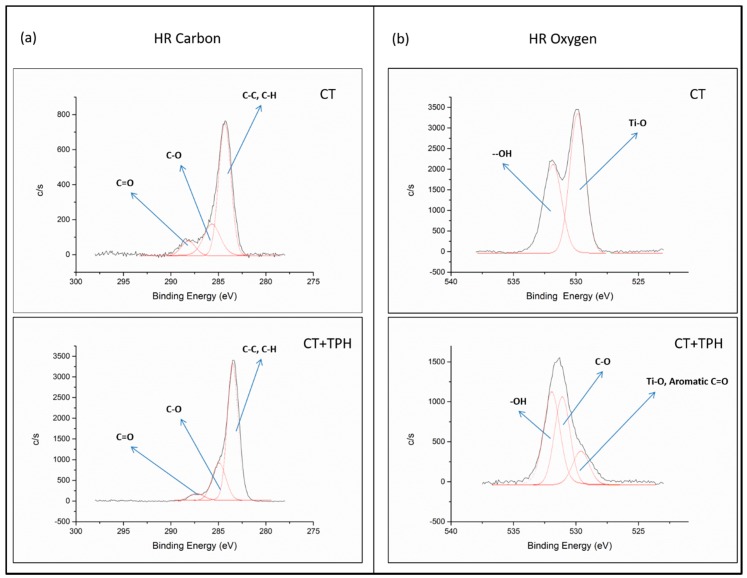
XPS high resolution (HR) spectra of (**a**) C1s and (**b**) O1s of sample CT and CT + TPH.

In order to detect the specific chemical groups exposed on the surfaces, high-resolution spectra of the carbon C1s and the oxygen O1s regions were recorded, as reported in Figure 1.

In the high-resolution spectra of carbon (Figure 1a) of both the samples, the peaks at 284.69 eV (CT sample) and 284.73 eV (CT + TPH sample) can be ascribed to C-C and C-H bonds [32]. There are another two peaks at 285.97 − 286.19 eV and 288.76 − 288.71 eV, which can be correlated to C-O and C=O groups, respectively [36,37,38]. A clear difference between the bare and the functionalized samples is visible in the oxygen region (Figure 1b).

For the CT samples, two peaks are present at 530.44 eV and at 532.49 eV and these can be correlated with the Ti-O bond of the titanium oxide and with the –OH groups exposed by the surface titanium oxide layer after the chemical treatment, respectively [33,34,39]. On the spectrum of the sample CT + TPH, there are three peaks at 530.08 eV, 531.05 eV and 532.81 eV. These peaks can be correlated with aromatic C=O [40], with C-O bonds and O=C-OH [39,40] and with –OH groups of the polyphenols [23,25,39], respectively.

### 2.2. Cell Culture Characterization

**Figure 2:**
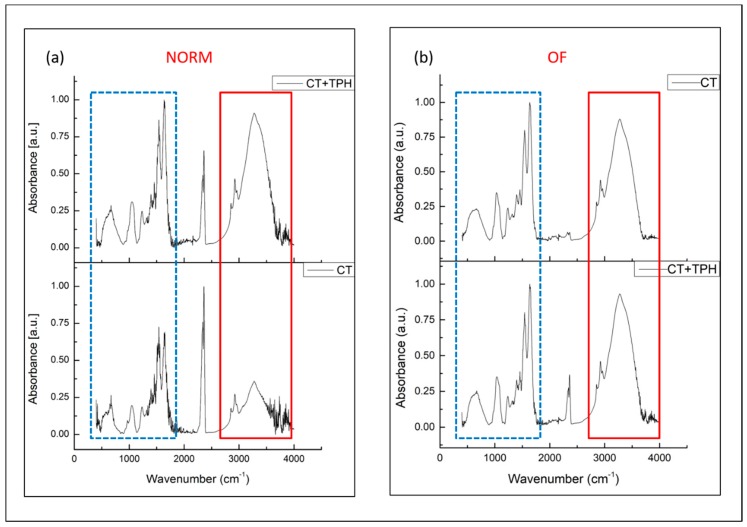
FITR spectra of the samples CT and CT + TPH cultured with (**a**) the normal medium, (**b**) the medium with the osteoinductive factors. Two zones of interest are highlighted with the blue rectangle between 400 and 1100 cm^−1^ and by the red rectangle between 2700 cm^−1^ and 3600 cm^−1^.

FTIR spectra acquired of the samples CT and CT + TPH, after the culture of KUSA A1 cells with the normal medium (NORM) or with the medium with the osteoinductive factors (OF), are reported in Figure 2a,b, respectively, in the range between 400 cm^−1^ and 4000 cm^−1^. Two significant regions are highlighted by the blue rectangle between 400 and 1100 cm^−1^ and by the red rectangle between 2700 cm^−1^ and 3600 cm^−1^. The peak around 2500 cm^−1^ is due to absorption of the atmospheric CO_2_ [41]. Signals from both protein vibrations and PO_4_^3−^ vibrations of hydroxyapatite (HAp) were detected between 400 and 760 cm^−1^ [38] (see the blue rectangle). The bands between 960 and 1100 cm^−1^ are due to the deposition of hydroxyapatite (Figure 2a) [42]. The bands at 1540 cm^−1^ and 1645 cm^−1^ are correlated with Amide I and II (1540 cm^−1^, 1645 cm^−1^) [43] and show the different behavior between the two different surfaces.

For the spectra of the samples CT and CT + TPH cultured with osteoinductive factors (Figure 2b), the signals in the zones of interest were still present, but there were no significant differences between the samples CT and CT + TPH.

**Figure 3:**
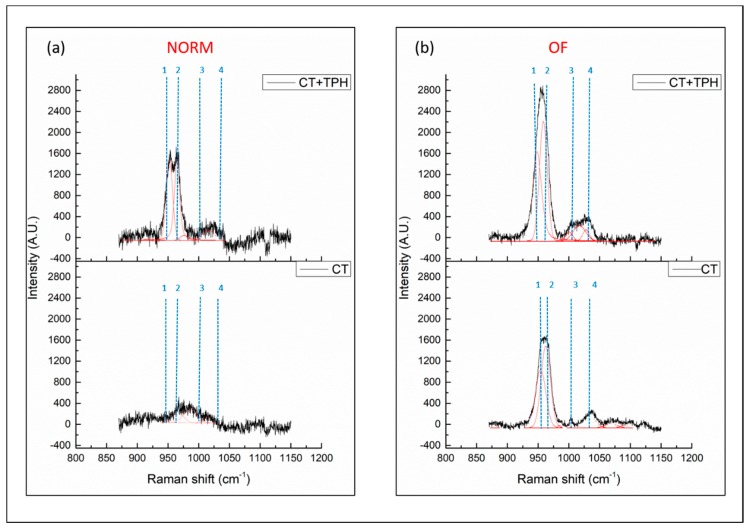
RAMAN spectra of samples CT and CT + TPH cultured with (**a**) the normal medium, (**b**) the medium with the osteoinductive factors.

Deposition of hydroxyapatite was also investigated by means of RAMAN spectroscopy and the results are reported in Figure 3.

The bands of interest, due to the vibration of hydroxyapatite, are between 900 and 1000 cm^−1^ [44,45]. In Figure 3a, for the samples cultured in the normal medium, bands are barely visible on the CT sample, while they are well present on the spectrum of the sample functionalized with polyphenols. It is also possible to deconvolute the bands into two peaks at 940 cm^−1^ and 960 cm^−1^, numbered as 1 and 2 in Figure 3, which are respectively correlated with the vibration of amorphous HAp and crystalline HAp [44,45,46,47].

Another important feature, clearly visible in the spectrum, is the presence/absence of phenylalanine bands at 1004 cm^−1^ [44] numbered as 3 in Figure 3. This is clearly visible in the samples treated with osteoinductive factors while it is absent or not clear in the others. Another interesting feature of the spectra concerns the band at 1035 cm^−1^ [44] numbered as 4, which is related to vibrational modes of phenylalanine and collagen compounds.

**Figure 4:**
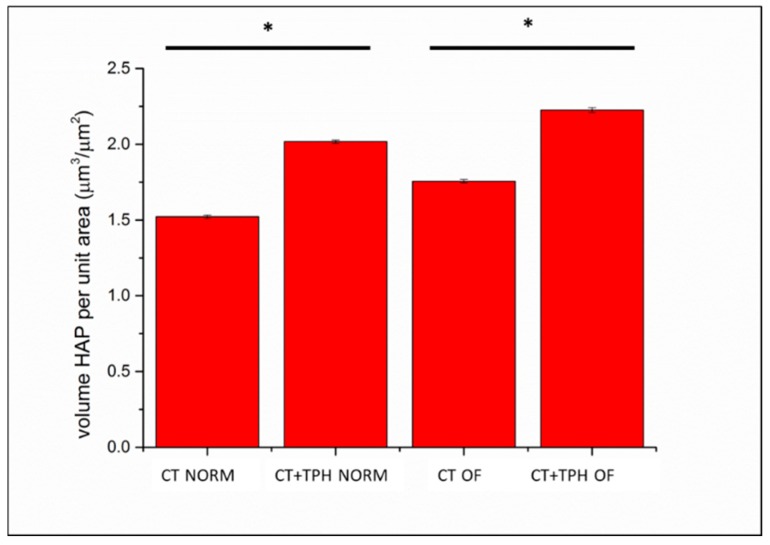
Extracellular matrix quantification on the surface of the samples CT and CT + TPH after the cell culture with the normal medium and with the osteoinductive factors (*p* < 0.01 (*)).

In Figure 4, the volume of the extracellular matrix detected on the surfaces of the samples cultured with the normal medium and with the osteoinductive factors is reported. The presence of the osteoinductive factors enhances the deposition of extracellular matrix for both the CT and CT+TPH samples, but with both types of culture, the presence of the polyphenols allows KUSA A1 to deposit a higher amount of extracellular matrix.

**Figure 5:**
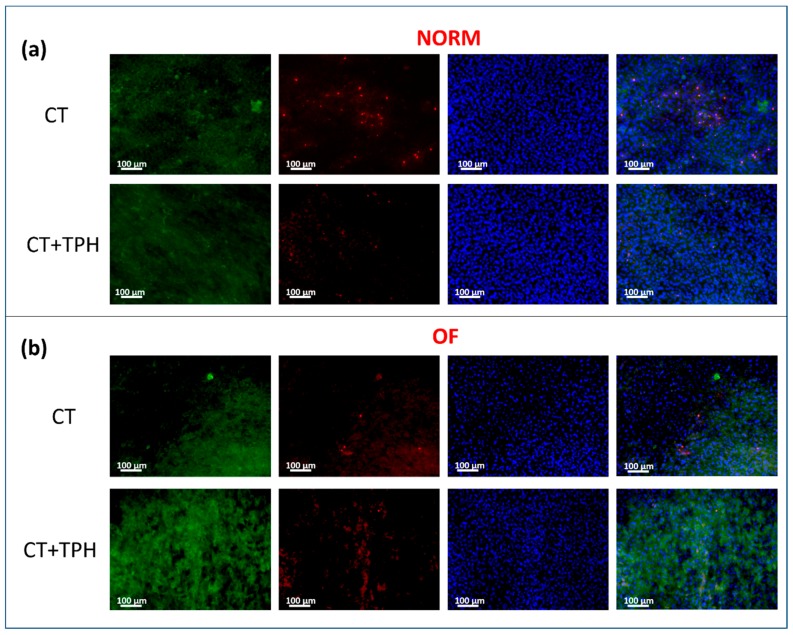
Fluorescence images performed using blue probe for cell nucleus (DAPI), Red probe for dead cells (PI) and green probe for osteocalcin (Hoechst33342). The images were performed at 20 × magnifications; (**a**) reports the results of the samples cultured with the normal medium and (**b**) shows the results of the medium cultured with the osteoinductive factors.

Figure 5 shows the fluorescence images of the samples cultured with KUSA A1. The analysis used a blue probe for cell nucleus (DAPI), red for dead cells (PI) and green for osteocalcin (Hoechst33342).

The nuclei of the cells are marked in blue and a high presence of cells is visible on all the samples. The green probe, which shows the presence of osteocalcin, highlights the differentiation of the KUSA A1 into osteoblasts and the deposition of hydroxyapatite by cells themselves. This protein is well present on the surface of the CT samples and it is deposited around the cells (Figure 5a). For the CT + TPH samples, the amount of osteocalcin is higher and it seems to continuously overlay the cells. In the case of the samples cultured with the osteoinductive media (Figure 5b), osteocalcin is well present, but it is accumulated in one zone in the case of the bare sample and well dispersed on the sample functionalized with polyphenols. Regarding the red probe, which marks the nuclei of the dead cells, a reduction of apoptosis is visible for the samples functionalized with polyphenols compared to the bare samples, in the case of the cultures both with and without the osteoinductive factors.

## 3. Discussion

### 3.1. Detection of Polyphenols

The photometric measurements with the Folin–Ciocalteu test confirm the presence of the polyphenols on the surface of the samples, as well as the maintenance of their redox activity after grafting, and the result is similar to the one found in a previous work regarding functionalization with gallic acid [48]. The formation of a continuous layer of TPH biomolecules grafted on the surface of the samples is also suggested by the XPS elemental analysis which showed a very low amount of Ti visible on the functionalized samples (Table 1). Accordingly, even if some carbon contamination is observed on CT samples, as widely reported in the literature for reactive titanium surface [34,35], the increase in carbon on CT + TPH samples can be associated with the presence of polyphenols grafted to the surfaces, as has been observed in a previous work [48].

The presence of a significant amount of oxygen on the surface of the CT samples is due to the presence of an oxide layer that is thicker than the native one (about 300 nm) and rich in –OH groups. For the functionalized samples, the much smaller amount of oxygen could be correlated with the presence of polyphenols on the outermost surface layer, in which the percentage of carbon is significantly higher than oxygen, instead of titanium oxide. The presence of calcium on the surface of the CT + TPH samples can be explained considering that TPH grafting has been performed in SBF solution (which contains Ca ions) and that polyphenols are able to bind calcium [23,49]. The Ca^2+^ ions are supposed to bridge the substrate and the grafted biomolecules, in fact, as supposed in a previous work [48], the grafted polyphenols form heterogeneous ternary complexes with Ca^2+^ ions. However, in this case the detectable amount of calcium is lower than in the previous work because of the larger size of the tea polyphenols used in this case with respect to gallic acid, which can hide calcium detection from XPS analyses.

The three peaks observed in the high-resolution spectrum of the carbon region (Figure 1a) can be attributed to the presence of surface contaminants on the CT sample, as mentioned above and which is widely reported in the literature concerning titanium surfaces [34,35]. On the other hand, for the CT + TPH sample, the same signals around 286 eV and 288 eV show a higher intensity with respect to C-C and C-H bonds and can be correlated to the presence of polyphenols. In fact, these molecules are rich in C-O and C=O bonds [23,25].

For the high-resolution spectrum of the oxygen region for the CT sample, two peaks are present; 530.44 eV, due to Ti-O bonds and 532.49 eV, due to Ti-OH bonds, and are abundantly exposed with the chemical treatment. For the CT + TPH samples it is possible to observe three peaks in Figure 1b: 530.08 eV, 531.05 eV and 532.81 eV. In this case the first contribution cannot be attributed only to the Ti-O, because of the very low amount of Ti detected on this sample (0.4% from the survey spectrum, Table 1), while it can be correlated also with aromatic C=O [40], which are abundant in polyphenols. The peak at 531.05 eV can be correlated with C-O bonds and O=C-OH [39,40] and the peak at 532.81 eV can be attributed to the –OH groups, which are not only present on the oxide layer, but are abundant in polyphenols [23,39,40]. Contrary to previous observations of bioactive glasses [23] no shift in the OH signal was detected after polyphenols grafting, as previously observed on gallic acid functionalized CT samples [48].

The results obtained by means of spectroscopic analysis with the Folin–Ciocalteu method and XPS confirm the success of the functionalization with the green tea polyphenols on the surface of the chemically treated titanium alloy.

### 3.2. Cell Culture Characterization

The FTIR analysis performed on samples after the cell cultures shows some differences between the cell behavior on CT and CT + TPH samples. In particular, for the spectra of the samples CT and CT + TPH cultured without osteoinductive factors, some clear differences can be observed. The bands between 960 and 1100 cm^−1^ (Figure 2a) due to the deposition of hydroxyapatite [44] are both slightly higher in the spectra of the CT + TPH samples in the case of the samples cultured in a normal medium (Figure 2a). Also, the bands of Amide I and II (1540 cm^−1^, 1645 cm^−1^) [43] show the different behavior of the two different surfaces. The samples functionalized with polyphenols have higher bands intensity indicating an increase of protein constituents (mainly type I collagen [50]). These features suggest that polyphenols increase the deposition of protein compounds of the collagen matrix. The bands in the red box between 2800–3000 cm^−1^ due to C-H stretching in proteins, vibrational modes of CH_2_ stretching of lipids related to collagen formation [44] and between 3000–3600 cm^−1^ due to the absorbance of O-H stretching in water and organic components [44] are clearly more intense for the samples functionalized with the polyphenols in the culture without osteoinductive factors. According to these two portions of the spectra, it can be concluded that the mesenchymal stem cells were differentiated into osteoblasts able to produce hydroxyapatite thanks to the stimulation of the substrate of the culture. Moreover, the effect of the polyphenols in increasing the production of collagen, mineralized and non-mineralized extracellular matrix is also evident [42,49,50,51,52,53]. For the spectra of the samples CT and CT + TPH cultured with osteoinductive factors (Figure 1b), no clear differences could be observed probably because the effect of polyphenols is hidden by the strong actions of the osteoinductive factors.

The Raman spectroscopy analyses performed in order to investigate the deposition of hydroxyapatite confirm the previous results obtained with FTIR for the cell culture without osteoinductive factors (Figure 3a) and suggest a higher deposition of hydroxyapatite on the CT + TPH sample. Also, observing the two peaks (1,2), CT + TPH has two peaks with the same intensity due to amorous and crystalline HAp, which highlights a comparable amount of amorphous and crystalline hydroxyapatite: however, in the case of the CT sample a significant deconvolution cannot be obtained because of the low intensity of the band due to a low deposition of HAp. In the case of the samples cultured with the osteoinductive factor, the band of hydroxyapatite is clearly visible for both the bare and the functionalized samples with the same intensity ratio (about 1.5) of crystalline/amorphous hydroxyapatite. Comparing the spectra of CT and CT+TPH, it can be observed that, for the CT + TPH sample the intensity of the band is higher. This phenomenon can be correlated with the polyphenols’ osteoinductive effect in synergy with the effect of the osteoinductive factors, in accordance with the behavior observed in the normal medium. The peak due to phenylalanine (1004 cm^−1^) [44] (Figure 3), numbered as 3, is commonly used as a cellular viability indicator. It is a little higher in the titanium alloy sample functionalized with polyphenol, confirming the FITR analysis and suggesting the positive influence of polyphenols on cell response. Also, the band numbered as 4 at 1035 cm^−1^, due to phenylalanine and collagen compounds [40], is clearly observed on the samples treated with osteoinductive factors and the signal is higher for the samples functionalized with polyphenols, again confirming that polyphenols improve cellular response and differentiation.

The analyses with laser microscope (Figure 4) of the amount of extracellular matrix produced by KUSA A1 on the surface of the samples of CT + TPH substrates compared to the CT substrates, also highlighted the stimulating effect that the polyphenols have on the cells.

Fluorescence microscopy observation of cells cultured on CT and CT + TPH samples after staining, evidence an up-regulation of osteocalcin (Figure 5). This up-regulation is in agreement with the literature [54]. It is reported, that this effect can be combined with promotion of differentiation of the osteoblast through the RunX2 transcription activity [55] and the upregulation of the related genes. As reported, the differentiated osteoblast show a higher expression of bone morphogenic proteins and alkaline phosphatase with an improvement in mineralization [6]. An increase in the production of collagen 1α1 is also possible [7] in a similar way as was observed in the FTIR analysis in Figure 2. Polyphenols showed the ability to improve mineralization and osteoblast differentiation, suggesting their potential application as osteopromotive factors while avoiding the side effects and the cost of other osteoinductive factors, such as grown factors [56].

The outcomes of the present report are promising, but in future, Randomized controlled trials are needed in order to confirm the in vitro results of the present report.

## 4. Materials and Methods

### 4.1. Polyphenols Extraction

Polyphenols used for functionalization were extracted from green tea leaves (Longjing, manufactured in Hangzhou, China). Extraction was performed as described in [23] with a conventional solvent extraction procedure in a solution of 80% ethanol in water (1 h at 60 °C and 120 rpm). The natural polyphenols extracted from green leaves were named TPH.

### 4.2. Surface Functionalization

Disks of Ti6Al4V alloy (2 mm thick, 10 mm in diameter, ASTM B348, Gr5, Titanium Consulting and Trading, Milan, Italy) were cut from a cylindrical bar with an automatic cutter (Struers Accutom 5, Struers, Ballerup, Denmark). The samples were polished with SiC abrasive paper up to 4000 grit. After polishing, the samples were washed for 5 min in acetone in an ultrasonic bath and then washed three times in ultrapure water for 5 min using an ultrasonic bath. After washing, the samples were dried at room temperature. In order to induce bioactive behavior, to make the surface suitable for the functionalization with a high presence of hydroxyl groups and to obtain a micro and nano-structured oxide on the surface (Sa ≅ 15 nm), a patented chemical treatment was applied [57]. The chemical treatment consisted of hydrofluoric acid etching and subsequent controlled oxidation in hydrogen peroxide [32,33]. After the chemical treatment and immediately before functionalization, the samples were exposed for 1h at UV irradiation in order to improve the chemical reactivity of the surface. The samples described above were named “chemical-treated” (CT) from now on. The CT samples were then functionalized in clean conditions with TPH, putting the samples in 5 mL of the solution of functionalization for 3 h at 37 °C in a holder covered with aluminum in order to avoid UV degradation of the molecules. Stock solution with concentration 1 mg/mL of solute was obtained dissolving 100 mg of TPH in 100 mL of simulated body fluid (SBF) solution prepared according to the protocol described by Kokubo [58] and stirred for 1 h. After 1 h, the solution was filtered with a 0.2 µm filter in order to avoid bacterial contamination. All the procedures were performed under a laminar flow cabinet (FASTER CYTOSAFE). The concentration of reactive polyphenols detected in the source solution of functionalization prepared with a 1 mg/mL concentration was 0.54 ± 0.01 mg/mL GAE equivalent. The extract was not purified and contains a mixture of polyphenols, but the presence of mineral salts is also possible.

After 3 h of functionalization, the samples were washed 2 times in sterile double distilled water, dried under laminar flow and then preserved in the dark into sterile holders. The functionalized samples were named CT + TPH.

### 4.3. Detection of the Polyphenols

The presence of the grafted green tea polyphenols was detected on the surfaces by means of spectrophotometric analysis (CARY 500 Varian, Agilent, Santa Clara, CA USA) with the Folin–Ciocalteu method and of X-ray photoelectron spectroscopy analysis (XPS, PHI 5000 Versaprobe, Physical Electronics, Chanhassen, MN, USA). The Folin–Ciocalteu method employed was adapted to solid samples as described in [25,59]. This method determines the redox reactivity of the phenolic compounds and obtains the amount of active molecules grafted on the surface of the samples. The samples were functionalized as described in [25]. The amount of polyphenols was quantified through a calibration curve obtained as described in [60]. This curve quantifies the amount of polyphenols in gallic acid equivalents [mg/mL] by using the fitting equations derived from data (y = 21.715x − 0.0088) with a coefficient of correlation R² = 0.9999. This analysis was performed in triplicate.

As far as XPS analyses are concerned, both survey and high-resolution spectra (carbon and oxygen regions) were performed in order to quantify the elements on the surface and to identify the characteristic chemical groups.

### 4.4. Cell Culture

Two different cell cultures were performed, one with a normal medium (NORM) and one with the addition of osteoinductive factors (OF). In both the cultures, the cells used were KUSA A1, cultured for one week on the sample surfaces with two changes of medium. The normal medium was composed of DMEM (D-glucose, L-glutamine, phenol red, and sodium pyruvate), 10% FBS (Fetal Bovine Serum) with SNP (sodium nitroprusside) (2%). The culture with the osteoinductive factor was performed with 50 mg/mL of ascorbic acid, 10 mM b-glycerol phosphate, 100 mM hydrocortisone, 10% FBS added to 4.5 g/L of glucose DMEM. After culture, the cells were fixed on the samples with 4% formaldehyde for FTIR, RAMAN and Laser microscope analysis.

For the fluorescent microscope, the protocol of immunostaining was applied after the culture. The disks were removed from the medium and washed twice in PBS, after they were fixed with 4% formaldehyde (for 15 min at room temperature) and washed again twice with in phosphate buffered saline solution (PBS). Then, the primary antibody was added and incubated at room temperature for 30 min and at the end washed twice in PSB. Then, the staining solution containing mixed Hoechst33342, PI and DAPI and secondary antibody Goat anti IgG FITC conjugate was added and incubated for 30 min at room temperature in the dark. Finally, the samples were again washed twice in PBS and dried at room temperature [59].

### 4.5. Cell Culture Characterization

Fourier Transform Infrared Spectroscopy (FTIR JASCO 4000, Jasco, Tokyo, Japan) analysis was performed on the surface of the CT and CT + TPH samples after the two different types of culture in order to investigate the viability and differentiation of the cells and the deposition of the hydroxyapatite in the presence of polyphenols.

RAMAN analysis (T-64000, Horiba/Jobin-Yvon, Kyoto, Japan) was carried out in order to investigate the presence and the different degree of crystallinity of hydroxyapatite on the samples with and without the polyphenols.

Laser microscope (Laser Microscope 3D & Profile measurements, Keyence, VK-x200 series, Osaka, Japan) analysis was performed in order to investigate and quantify the growth of the extracellular matrix on the surface of the samples. A map of all the samples was taken at 10× and an analysis of the surface volume was conducted on single images. Data were analyzed with the software of the machine (VK analyzer, Keyence Ltd. HQ & Laboratories, Osaka, Japan). Fluorescence microscopy (BZ-X700; Keyence, Osaka, Japan) analysis was performed in order to understand cell viability and osteocalcin production with or without polyphenols. Images were collected at 20× magnification using three different probes: blue for cell nucleus (DAPI), red for dead cells (PI) and green for osteocalcin (Hoechst33342).

## 5. Conclusions

Green tea polyphenols were successfully grafted to the surface of a chemically-treated titanium alloy and their effect as cell stimulus was tested with the final aim of coupling inorganic and biological mineralization abilities. The presence and activity of the grafted polyphenols on the surfaces were observed and after cultures of mesenchymal stem cells, with or without the addition of osteoinductive factors, the surfaces were analyzed. The analysis showed the presence of hydroxyapatite on all the cultures, suggesting the differentiation of the cells into osteoblasts, and an increased presence of collagen and HAp on the samples functionalized with polyphenols.

We can conclude that natural polyphenols are promising molecules that are able to promote osteoblast differentiation and mineralization without the high costs of other osteoinductive factors. The use of these molecules for functionalization of biomaterials surfaces seems to be an effective way for in loco administration and overcoming the problem of dosage due to the digestive metabolism if taken orally.

## Figures and Tables

**Table 1:** The atomic percentages of the elements detected on the surface of samples CT + TPH and CT by X-ray photoelectron spectroscopy (XPS) survey analyses (Uncertainty of measurements 0.1–0.2% at).

Elements [% at]	Samples	
	CT	CT+TPH
C	20.7	76.6
O	60.7	20.3
P	-	0.9
Ca	-	0.5
N	2.3	0.8
F	-	0.5
Ti	16.3	0.4

## References

[1] Chen D.I., Dou Q.P. (2008). Tea polyphenols and their roles in cancer prevention and chemotherapy. Int. J. Mol. Sci..

[2] Ferruzzi M.G. (2010). The influence of beverage composition on delivery of phenolic compounds from coffee and tea. Physiol. Behav..

[3] Rusak G., Komes D., Likić S., Horžić D., Kovač M. (2008). Phenolic content and antioxidative capacity of green and white tea extracts depending on extraction conditions and the solvent used. Food Chem..

[4] Carloni P., Tiano L., Padella L., Bacchetti T., Customu C., Kay A., Damiani E. (2013). Antioxidant activity of white, green and black tea obtained from the same tea cultivar. Food Res. Int..

[5] Bancirova M. (2010). Comparison of the antioxidant capacity and the antimicrobial activity of black and green tea. Food Res. Int..

[6] Srivastava S., Bankar R., Roy P. (2013). Assessment of the role of flavonoids for inducing osteoblast differentiation in isolated mouse bone marrow derived mesenchymal stem cells. Phytomedicine.

[7] Vester H., Holzer N., Neumaier M., Lilianna S., Nüssler A.K., Seeliger C. (2014). Green Tea Extract (GTE) improves differentiation in human osteoblasts during oxidative stress. J. Inflamm..

[8] Scalbert A., Morand C., Manach C., Rémésy C. (2002). Absorption and metabolism of polyphenols in the gut and impact on health. Biomed. Pharmacother..

[9] Correa-Betanzo J., Allen-Vercoe E., McDonald J., Schroeter K., Corredig M., Paliyath G. (2014). Stability and biological activity of wild blueberry (Vaccinium angustifolium) polyphenols during simulated in vitro gastrointestinal digestion. Food Chem..

[10] Galić A., Dragović-Uzelac V., Levaj B., Bursać Kovačević D., Pliestić S., Arnautović S. (2009). The polyphenols stability, enzyme activity and physico-chemical parameters during producing wild elderberry concentrated juice. Agric. Conspec. Sci..

[11] Kuhnle A.R., Bremner G., Hubbard P., Moore G.P., Rice-Evans C.A. (2002). The metabolic fate of dietary polyphenols in humans. Free Radic. Biolog. Med..

[12] Sileika T.S., Barrett D.G., Zhang R., Lau K.H.A., Messersmith P.B. (2013). Colorless multifunctional coatings inspired by polyphenols found in tea, chocolate, and wine. Angew. Chem. Int. Ed. Engl..

[13] Berlier G., Gastaldi L., Sapino S., Miletto I., Bottinelli E., Chirio D., Ugazio E. (2013). MCM-41 as a useful vector for rutin topical formulations: Synthesis, characterization and testing. Int. J. Pharm..

[14] Wang K., Wu Y., Li H., Li M., Zhang D., Feng H., Fan H. (2013). Dual-functionalization based on combination of quercetin compound and rare earth nanoparticle. J. Rare Earths.

[15] Mohanty R., Thennarasu S., Mandal A.B. (2014). Resveratrol stabilized gold nanoparticles enable surface loading of doxorubicin and anticancer activity. Colloids. Sur. B Biointerfaces.

[16] Saikia J.P., Konwarh R., Konwar B.K., Karak N. (2013). Isolation and immobilization of Aroid polyphenol on magnetic nanoparticles: Enhancement of potency on surface immobilization. Colloids. Sur. B Biointerfaces.

[17] Džunuzović E.S., Džunuzović J.V., Marinković A.D., Marinović-Cincović M.T., Jeremić K.B., Nedeljković J.M. (2012). Influence of surface modified TiO2 nanoparticles by gallates on the properties of PMMA/TiO2 nanocomposites. Eur. Polym. J..

[18] Gurzawska K., Svava R., Yihua Y., Haugshøj K.B., Dirscherl K., Levery S.B., Jørgensen N.R. (2014). Osteoblastic response to pectin nanocoating on titanium surfaces. Mater. Sci. Eng. C Mater. Biol. Appl..

[19] Mohan L., Anandan C., Rajendran N. (2016). Drug release characteristics of quercetin-loaded TiO2 nanotubes coated with chitosan. Int. J. Biol. Macromol..

[20] Erakovic S., Jankovic A., Tsui G.C., Tang C.Y., Miskovic-Stankovic V., Stevanovic T. (2014). Novel bioactive antimicrobial lignin containing coatings on titanium obtained by electrophoretic deposition. Int. J. Mol. Sci..

[21] Córdoba A., Satué M., Gómez-Florit M., Monjo M., Morey J.M.R. (2015). Flavonoid coated Titanium surfaces for Bioactive Bone implants. Stem Cell Transl. Investig..

[22] Cazzola M., Vernè E., Cochis A., Sorrentino R., Azzimonti B., Prenesti E., Ferraris S. (2017). Bioactive glasses functionalized with polyphenols: In vitro interactions with healthy and cancerous osteoblast cells. J. Mater. Sci..

[23] Cazzola M., Corazzari I., Prenesti E., Bertone E., Vernè E., Ferraris S. (2016). Bioactive glass coupling with natural polyphenols: Surface modification, bioactivity and anti-oxidant ability. Appl. Sur. Sci..

[24] Zhang X., Ferraris S., Prenesti E., Verné E. (2013). Surface functionalization of bioactive glasses with natural molecules of biological significance, part II: Grafting of polyphenols extracted from grape skin. Appl. Sur. Sci..

[25] Zhang X., Ferraris S., Prenesti E., Verné E. (2013). Surface functionalization of bioactive glasses with natural molecules of biological significance, Part I: Gallic acid as model molecule. Appl. Surf. Sci..

[26] Ferraris S., Zhang X., Prenesti E., Corazzari I., Turci F., Tomatis M., Vernè E. (2016). Gallic acid grafting to a ferrimagnetic bioactive glass-ceramic. J. Non. Cryst. Solids.

[27] Tolga Suer T.B., Yaman Z., Buyuksarac B. (2016). Correlation of Fractal Dimension Values with Implant Insertion Torque and Resonance Frequency Values at Implant Recipient Sites. Int. J. Oral. Maxillofac. Implants.

[28] Suer B.T., Kocyigit I.D., Kaman S., Tuz H.H., Tekin U., Atil F. (2014). Biomechanical evaluation of a new design titanium miniplate for the treatment of mandibular angle fractures. Int. J. Oral. Maxillofac. Surg..

[29] Scribante A., Montasser M.A., Radwan E.S., Bernardinelli L., Alcozer R., Gandini P., Sfondrini M.F. (2018). Reliability of Orthodontic Miniscrews: Bending and Maximum Load of Different Ti-6Al-4V Titanium and Stainless Steel Temporary Anchorage Devices (TADs). Materials.

[30] Oldani C., Dominguez A. (2012). Titanium as a Biomaterial for Implants. Recent Advances in Arthroplasty.

[31] Long M., Rac H.J. (1998). Titanium alloys in total joint replacement—A materials science perspective. Biomaterials.

[32] Ferraris S., Spriano S., Bianchi C.L., Cassinelli C., Vernè E. (2011). Surface modification of Ti-6Al-4 V alloy for biomineralization and specific biological response: Part II, alkaline phosphatase grafting. J. Mater. Sci. Mater. Med..

[33] Ferraris S., Spriano S., Pan G., Venturello A., Bianchi C.L., Chiesa R., Faga M.G., Maina G., Verne E. (2011). Surface modification of Ti–6Al–4V alloy for biomineralization and specific biological response: Part, I., inorganic modification. J. Mater. Sci. Mater. Med..

[34] Textor M., Sittig C., Frauchiger V., Tosatti S., Brunette D.M. (2001). Properties and biological significance of natural oxide films on titanium and its alloys. Titanium in Medicine.

[35] Morra M., Cassinelli C., Bruzzone G., Carpi A., Santi G.D., Giardino R., Fini M. (2003). Surface chemistry effects of topographic modification of titanium dental implant surfaces: 1. Surface analysis. Int. J. Oral. Maxillofac. Implants.

[36] Qiao G., Su J., He M. (2012). Effect of (−)-epigallocatechin gallate on electrochemical behavior and surface fi lm composition of Co-Cr alloy used in dental restorations. Dent. Mater. J..

[37] Moulder J.F., Stickle W.F., Sobol P.E., Bomben K.D. (1992). Handbook of X-ray Photoelectron Spectroscopy: A reference Book of Standard Spectra for Identification and Interpretation of XPS Data.

[38] (2000). NIST X-ray Photoelectron Spectroscopy Database, NIST Standard Reference Database Number 20.

[39] Oxygen 1s for Organic Compounds. http://www.xpsfitting.com/2013/08/oxygen-1s-for-organic-compounds.html.

[40] Lee H.P., Lin D.J., Yeh M.L. (2017). Phenolic modified ceramic coating on biodegradable Mg alloy: The improved corrosion resistance and osteoblast-like cell activity. Materials.

[41] Movasaghi Z., Rehman S., ur Rehman D.I. (2008). Fourier transform infrared (FTIR) spectroscopy of biological tissues. Appl. Spectrosc. Rev..

[42] ur Rehman I., Movasaghi Z., Rehman S. (2012). Vibrational Spectroscopy for Tissue Analysis.

[43] Movasaghi Z., Rehman S., Rehman I.U. (2007). Raman spectroscopy of biological tissues. Appl. Spectrosc. Rev..

[44] Pully V.V. (2010). From cells to bone: Raman microspectroscopy of the mineralization of stromal cells. Anal. Chem..

[45] Hashimoto A., Chiu L.D., Sawada K., Ikeuchi T., Fujita K., Takedachi M., Yamagouchi Y., Kawata S., Murakami S., Tamiya E. (2014). In situ Raman imaging of osteoblastic mineralization. J. Raman Spectrosc..

[46] Pezzotti G., Zhu W., Boffelli M., Adachi T., Ichioka H., Yamamoto T., Marunaka Y., Kanamura N. (2015). Vibrational algorithms for quantitative crystallographic analyses of hydroxyapatite-based biomaterials: I., theoretical foundations. Anal. Bioanal. Chem..

[47] Cazzola M. (2018). Multifunctional surfaces for implants in bone contact applications. Ph.D. Thesis.

[48] Ejima H., Richardson J.J., Liang K., Best J.P., van Koeverden M.P., Such G.K., Cui J., Caruso F. (2013). One-step assembly of coordination complexes for versatile film and particle engineering. Science.

[49] Boskey A., Camacho N.P. (2007). FT-IR imaging of native and tissue-engineered bone and cartilage. Biomaterials.

[50] Woess C., Unterberger S.H., Roider C., Ritsch-Marte M., Pemberger N., Cemper-Kiesslich J., Pallua J.D. (2017). Assessing various Infrared (IR) microscopic imaging techniques for post-mortem interval evaluation of human skeletal remains. PLoS ONE.

[51] Koutsopoulos S. (2002). Synthesis and characterization of hydroxyapatite crystals: A review study on the analytical methods. J. Biomed. Mater. Res..

[52] Berzina-Cimdina L., Borodajenko N. (2012). Research of calcium phosphates using Fourier transform infrared spectroscopy. Infrared Spectroscopy-Materials Science Engineering and Technology.

[53] Al Mamun M.A., Hosen M.J., Islam K., Khatun A., Alam M.M., Al-Bari M.A.A. (2015). Tridax procumbens flavonoids promote osteoblast differentiation and bone formation. Biol. Res..

[54] Léotoing L., Davicco M.J., Lebecque P., Wittrant Y., Coxam V. (2014). The flavonoid fisetin promotes osteoblasts differentiation through Runx2 transcriptional activity. Mol. Nutr. Food Res..

[55] James A.W., LaChaud G., Shen J., Asatrian G., Nguyen V., Zhang X., Soo C. (2016). A review of the clinical side effects of bone morphogenetic protein-2. Tissue Eng. Part. B Rev..

[56] Spriano S., Vernè E., Ferraris S. (2010). Multifunctional Titanium Surfaces for Bone Integration. European Patent.

[57] Kokubo T. (1991). Bioactive glass ceramics: Properties and applications. Biomaterials.

[58] Singleton V.L., Orthofer R., Lamuela-Raventós R.M. (1999). Analysis of total phenols and other oxidation substrates and antioxidants by means of folin-ciocalteu reagent. Methods in Enzymology.

[59] Singh S.R. (2012). Somatic Stem Cells: Methods and Protocols.

[60] Köck E.M., Kogler M., Bielz T., Klötzer B., Penner S. (2013). In situ FT-IR spectroscopic study of CO_2_ and CO adsorption on Y_2_O_3_, ZrO_2_, and yttria-stabilized ZrO_2_. J. Phys. Chem. C Nanomater. Interfaces.

